# Activation of the galanin receptor 2 in the periphery reverses nerve injury-induced allodynia

**DOI:** 10.1186/1744-8069-7-26

**Published:** 2011-04-16

**Authors:** Richard P Hulse, David Wynick, Lucy F Donaldson

**Affiliations:** 1 Schools of Physiology and Pharmacology, University of Bristol, University Walk, Bristol, BS8 1TD, UK; 2 Clinical Sciences, University of Bristol, University Walk, Bristol, BS8 1TD, UK

## Abstract

**Background:**

Galanin is expressed at low levels in the intact sensory neurons of the dorsal root ganglia with a dramatic increase after peripheral nerve injury. The neuropeptide is also expressed in primary afferent terminals in the dorsal horn, spinal inter-neurons and in a number of brain regions known to modulate nociception. Intrathecal administration of galanin modulates sensory responses in a dose-dependent manner with inhibition at high doses. To date it is unclear which of the galanin receptors mediates the anti-nociceptive effects of the neuropeptide and whether their actions are peripherally and/or centrally mediated. In the present study we investigated the effects of direct administration into the receptive field of galanin and the galanin receptor-2/3-agonist Gal2-11 on nociceptive primary afferent mechanical responses in intact rats and mice and in the partial saphenous nerve injury (PSNI) model of neuropathic pain.

**Results:**

Exogenous galanin altered the responses of mechano-nociceptive C-fibre afferents in a dose-dependent manner in both naive and nerve injured animals, with low concentrations facilitating and high concentrations markedly inhibiting mechano-nociceptor activity. Further, use of the galanin fragment Gal2-11 confirmed that the effects of galanin were mediated by activation of galanin receptor-2 (GalR2). The inhibitory effects of peripheral GalR2 activation were further supported by our demonstration that after PSNI, mechano-sensitive nociceptors in galanin over-expressing transgenic mice had significantly higher thresholds than in wild type animals, associated with a marked reduction in spontaneous neuronal firing and C-fibre barrage into the spinal cord.

**Conclusions:**

These findings are consistent with the hypothesis that the high level of endogenous galanin in injured primary afferents activates peripheral GalR2, which leads to an increase in C-fibre mechanical activation thresholds and a marked reduction in evoked and ongoing nociceptive responses.

## Introduction

The neuropeptide galanin is expressed at low levels in ~5% of small diameter neurons in the intact adult rodent dorsal root ganglion (DRG) [1-3]. Higher levels of the peptide are also detected in the primary afferent terminals of the spinal cord (lamina II), the dorsal horn inter-neurons [4], and in a number of brain regions known to modulate nociception, including the arcuate nucleus and periaqueductal grey (PAG) [5,6]. After nerve injury and models of neuropathic pain, galanin expression is markedly increased in 30-40% of sensory neurons [7,8] and in the primary afferent terminals in the superficial layers of the dorsal horn [9].

Behavioural studies have demonstrated that intrathecal (i.t.) administration of galanin modulates nociception in a dose-dependent manner, with facilitation of nociceptive reflexes at low concentrations of galanin [10,11] and a striking inhibition at higher concentrations [12,13]. The anti-nociceptive effect of high dose galanin is enhanced following peripheral nerve injury [14,15]. Consistent with the central effects of galanin, subcutaneous injection of galanin into the receptive fields of wide dynamic range dorsal horn neurons (lamina V) inhibited noxious mechanically evoked activity in intact and nerve injured rats [15]. The anti-nociceptive effects of galanin are further substantiated by a number of different lines of galanin over-expressing (Gal-OE) transgenic mice which all have an increase in withdrawal thresholds in the intact state and/or a reduction in allodynia after nerve injury [16,17].

The observed anti-nociceptive effects of high levels of endogenous or exogenous galanin are mediated by the activation of one or more G-protein coupled galanin receptors (GalR1, GalR2 and GalR3). Studies using in-situ hybridization have shown that GalR1 and GalR2 mRNAs are expressed by 51% and 83% of adult rat DRG neurons, respectively [18]. The levels of both sub-types decrease after axotomy [19-21]. High levels of GalR1 are observed in the spinal inter-neurons in the dorsal horn of the spinal cord with no change after axotomy [22]. In contrast, there is very little GalR2 mRNA in the spinal inter-neurons in the dorsal horn [22,23]. Expression of GalR3 in the rodent spinal cord and DRG is very low as determined by RT-PCR [21,24], and undetectable using in-situ hybridisation [25].

The short half-life of galanin and Gal2-11 (a peptide fragment of galanin with 500-fold selectivity for GalR2 and GalR3, compared to GalR1 [26,27]) in plasma of less than 10 minutes [28,29], and the absence of high-affinity galanin receptor-specific pharmacological tools have largely precluded a detailed analysis of the roles played by each receptor subtype in nociception. A previous study using i.t. infusions of galanin or Gal2-11 in a rat neuropathic pain model concluded that the central anti-nociceptive effects of galanin are likely to be mediated by activation of GalR1 [26], consistent with the very low levels of GalR2 in the dorsal horn [22]. However, GalR1-KO mice have only minor changes in neuropathic pain in two different models [30,31]. Further, the recently described developmental deficits in the DRG of GalR2-KO mice [21] precludes an analysis of the nociceptive role played by GalR2 in the adult animal. To date, the nociceptive phenotype of a GalR3-KO mouse has not yet been published.

In summary, it has not been possible to directly analyse whether the anti-nociceptive actions of galanin are principally peripherally and/or centrally mediated, nor to dissect the roles played by each galanin receptor subtype in nociceptive processing in the adult animal. The aim of the present study was to test the hypothesis that peripheral activation of GalR2 can modulate nociceptive C-fibre afferent mechanical responses in naive rats and mice and that these effects are altered in the PSNI model of neuropathic pain. Here we show that administration of low concentrations of galanin and Gal2-11 directly into the receptive field of intact but injured sensory afferents facilitates, and high concentrations markedly inhibit mechano-nociceptor activity. Further, mechano-sensitive nociceptors in Gal-OE mice had significantly higher thresholds after PSNI than in WT mice, associated with an abolition of spontaneous neuronal firing, the neural correlate of ongoing pain.

These findings are consistent with the hypothesis that the high level of endogenous galanin in injured primary afferents activates peripheral GalR2, which leads to an increase in C-fibre mechanical activation thresholds and a marked reduction in evoked and ongoing nociceptive responses. This would be expected to lead to a decrease in C-fibre barrage into the spinal cord, which in turn would reduce central sensitisation and allodynia.

## Results

### Nociceptive effects of galanin are mediated via activation of GalR2 in intact rats and mice

**Figure 1:**
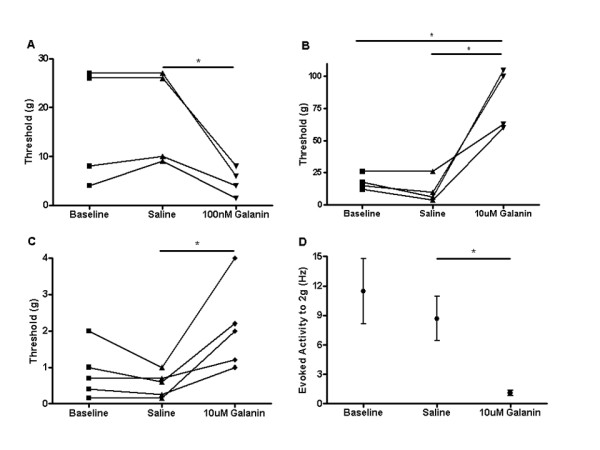
**Responses of mechano-C fibre nociceptors following peripheral administration of galanin in rat and mouse**. Subcutaneous injections of galanin were administered locally into the receptive field of mechanical nociceptive C-fibre afferents in intact naïve rodents. [A] 100 nM galanin significantly reduced mechanical activation thresholds in rats compared to vehicle (one way ANOVA with post-hoc Bonferroni multiple comparison test, *p < 0.05, n = 4). [B] 10 uM galanin, significantly increased mechanical thresholds in rats compared to vehicle (one way ANOVA with post-hoc Bonferroni multiple comparison test, *p < 0.05, n = 4). [C] 10 uM galanin significantly increased mechanical activation thresholds in mice (one way ANOVA, *p < 0.05, n = 5) and [D] 10 uM galanin significantly attenuated mechanically evoked activity to a 2 g von Frey hair in mice (one way ANOVA with post-hoc Bonferroni multiple comparison test, * p < 0.05, n = 5).

**Figure 2:**
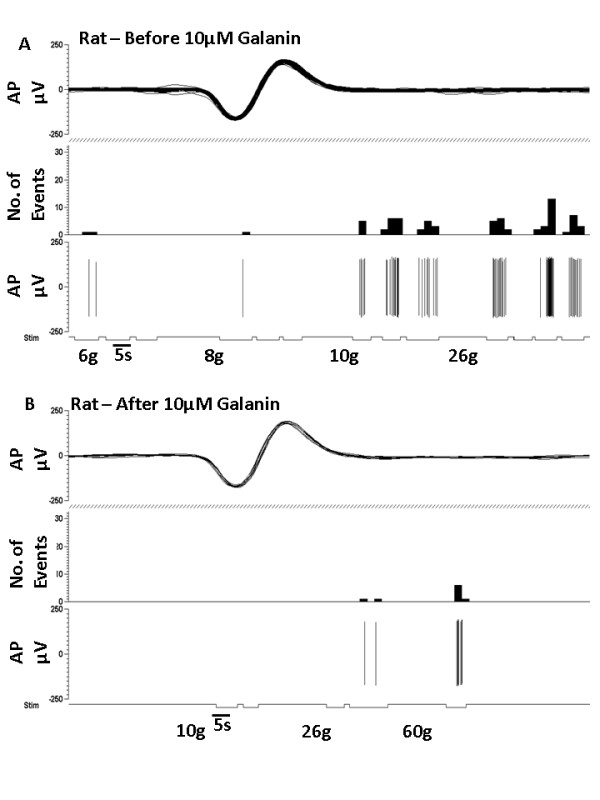
**Representative traces of a rat mechano-C fibre nociceptor in response to 10 μM galanin**. [A] C fibre mechanically sensitive nociceptor that responds to a 10 g von Frey hair. [B] Subcutaneous injection of 10 μM galanin into the receptive field led to an increase in mechanical activation threshold to 60 g.

We first studied whether administration of galanin into the peripheral receptive field modulates neuronal activity of mechanically sensitive cutaneous primary afferents. We also tested whether these effects were dose-dependent, using doses similar to those previously described to cause facilitation and suppression by i.t. administration [32]. In adult intact naïve rats, subcutaneous (s.c.) injection of 10 nM galanin into the peripheral receptive field of C-fibre nociceptors had no effect on the threshold of these fibres (data not shown). However, 100 nM galanin lowered mechanical activation thresholds (*p < 0.05, saline 18.0 ± 4.62 g vs galanin 4.5 ± 1.26 g, Figure 1A). In contrast, 10 uM galanin s.c. led to a striking increase in mechanical threshold in C-fibre mechano-nociceptors (*p < 0.01, saline 17.75 ± 2.75 g vs galanin 47.5 ± 12.50 g, Figure 1B and Figure 2A &2B). Of note, 10 uM galanin did not alter mechanical thresholds in low threshold mechanically sensitive A-beta fibres, (CV = 22.5-28 m/s) in the intact rat (baseline 0.9333 ± 0.53 vs saline 0.9333 ± 0.53 vs 10 uM galanin 1.133 ± 0.47, NS).

**Figure 3:**
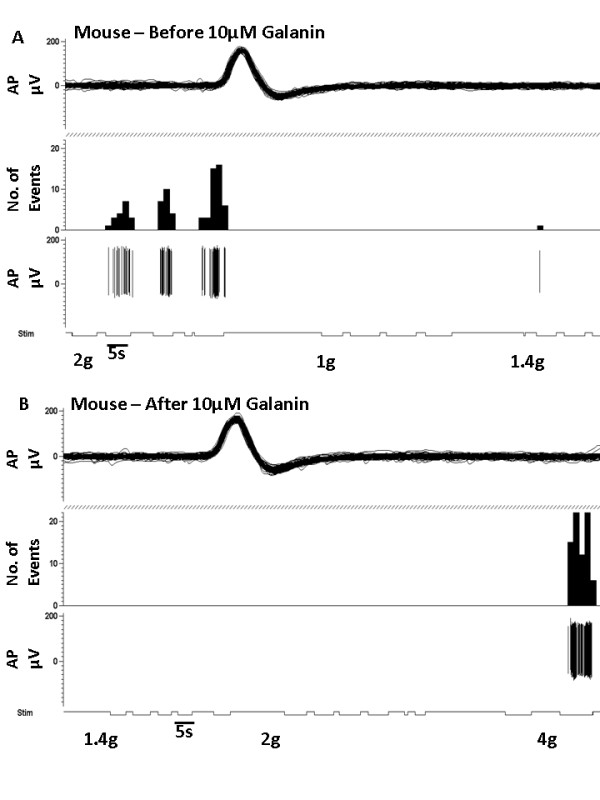
**Representative traces of a mouse mechano-C fibre nociceptor in response to 10 μM galanin**. [A] C fibre mechanically sensitive nociceptor that responds to a 2 g von Frey hair. [B] Subcutaneous injection of 10 μM galanin into the receptive field led to an increase in mechanical activation threshold to 4 g.

We then confirmed that similar responses to galanin in mechanically sensitive C-fibre afferents were also present in mice. In adult intact naive CBA/Bl6 mice 10 uM galanin was injected subcutaneously into the peripheral receptive field of C-fibre nociceptors. This increased mechanical thresholds (*p < 0.05, baseline 0.9 ± 10.32 g vs galanin 20 ± 0.55 g, Figure 1C and Figure 3A &3B) and reduced mechanically evoked activity (*p > 0.05, 2 g saline 8.66 ± 5.06Hz vs galanin 1.09 ± 0.63Hz, Figure 1D). As with the rat, 10 uM galanin s.c. had no effect on mechanical thresholds of A-beta fibres (CV = 12.5-18 m/s) in the adult intact naïve mouse (baseline 0.34 ± 0.06 g vs galanin 0.28 ± 0.07 g, NS).

**Figure 4:**
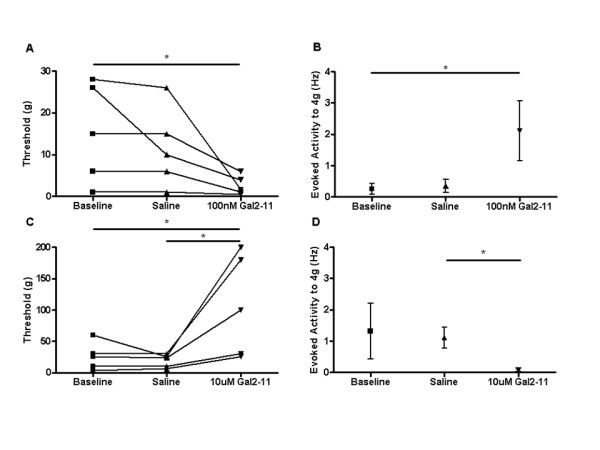
**Mechanical responses of C fibre nociceptors following GalR2 activation**. Subcutaneous injections of Gal2-11 were administered locally into the receptive field of mechanical nociceptive C-fibre afferents in intact naïve rats. [A] 100 nM Gal2-11 significantly decreased activation thresholds (one way ANOVA with post-hoc Bonferroni multiple comparison test, * p < 0.05, n = 4), and [B] facilitated mechanically evoked activity to a 4 g von Frey hair (one way ANOVA with post-hoc Bonferroni multiple comparison test, * p < 0.05, n = 4). [C] 10 uM Gal2-11 significantly increased mechanical activation thresholds (one way ANOVA with post-hoc Bonferroni multiple comparison test, * p < 0.05, n = 5), and [D] significantly decreased mechanically evoked activity to a 4 g von Frey hair (one way ANOVA with post-hoc Bonferroni multiple comparison test, *p < 0.05, n = 5).

We next used the GalR2/3 agonist, Gal2-11 to test whether the above responses to galanin were mediated via activation of GalR2. As discussed above, the expression of GalR3 mRNA in the DRG and dorsal horn is undetectable by in-situ hybridization and we therefore reasoned that the observed responses to Gal2-11 must be attributable to GalR2 activation. Identical doses of Gal2-11 to those used with galanin were directly applied to the peripheral receptive fields of C-fibre nociceptors in adult intact naïve rats. 100 nM Gal2-11 s.c. reduced mechanical activation thresholds (*p < 0.05, baseline 18.25 ± 4.83 g vs Gal2-11 3.10 ± 1.17 g, Figure 4A) and increased mechanically evoked activity (*p < 0.05, 4 g saline 0.35 ± 0.21Hz vs Gal2-11 2.12 ± 0.96Hz, Figure 4B). In contrast, 10 uM Gal2-11 s.c. markedly increased mechanical activation thresholds (*p < 0.05, baseline 25.2 ± 9.73 g vs Gal2-11 102.4 ± 34.44 g, Figure 4C) and decreased mechanically evoked activity (*p < 0.05, 4 g saline 1.15 ± 0.33Hz vs Gal2-11 0.088 ± 0.06Hz, Figure 4D). These findings are very similar to those observed with galanin and imply that the effects of galanin are largely dependent upon activation of GalR2.

### Nociceptive responses to galanin in the PSNI model of neuropathic pain

We then investigated whether the above dose-dependent effects of peripherally administered galanin and Gal2-11 were maintained in a model of neuropathic pain. We and others have shown that partial ligation of the saphenous nerve (PSNI) in rats and mice induces a robust increase in mechanical allodynia three days after saphenous nerve injury which lasts for at least 14 days [33,34], and induces a marked upregulation in galanin expression in the L3 and L4 DRG [33]. The simplicity of the PSNI model due to the superficial position of the saphenous nerve, minimally invasive surgery and the ability to easily record from intact (ie non-axotomised) nociceptive afferents has made it an attractive model to study injured primary afferents in an almost pure sensory nerve [33,34].

**Figure 5:**
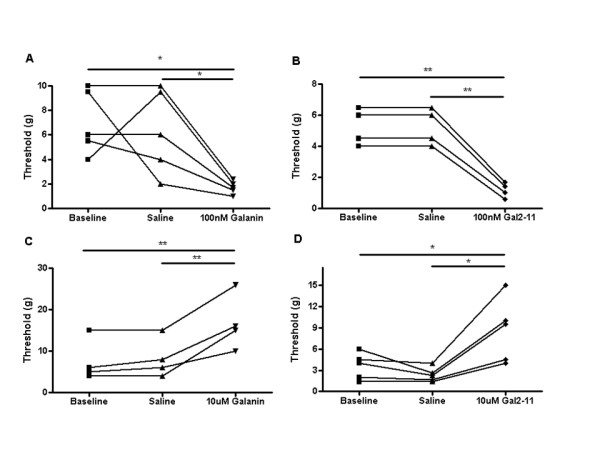
**C fibre nociceptor mechanical responses after galanin and Gal2-11 treatment in a model of neuropathic pain**. In PSNI (day three) nerve injured rats, 100 nM galanin and Gal2-11 s.c. enhanced mechanical activity in nociceptive afferents, whereas 10 uM galanin and Gal2-11 s.c. inhibited mechanical activity. [A] 100 nM galanin significantly reduced mechanical thresholds (one way ANOVA with post-hoc Bonferroni multiple comparison test, *p < 0.05, n = 5), and similarly [B] 100 nM Gal2-11 significantly reduced mechanical thresholds (one way ANOVA with post-hoc Bonferroni multiple comparison test, ** p < 0.01, n = 3). [C] 10 uM galanin significantly increased mechanical activation thresholds (one way ANOVA with post-hoc Bonferroni multiple comparison test, **p < 0.01, n = 4), and similarly [D] 10 uM Gal2-11 significantly increased mechanical activation thresholds (one way ANOVA with post-hoc Bonferroni multiple comparison test, * P < 0.05, n = 5).

**Figure 6:**
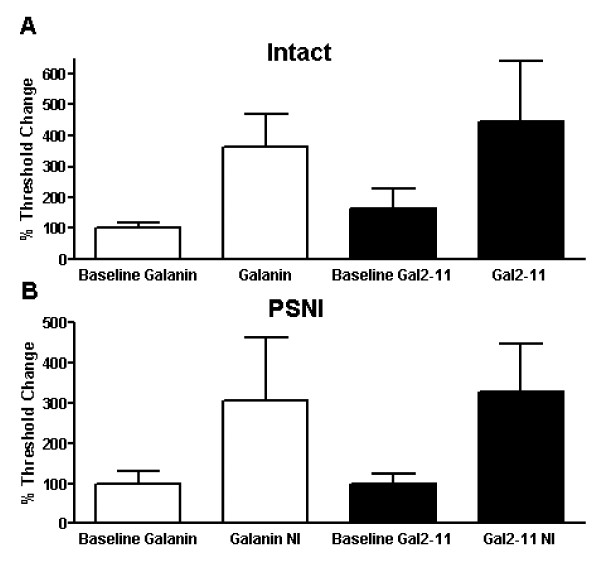
**The effects of 10uM galanin and 10uM Gal2-11 on nociceptive C-fibres in intact and nerve injured rats**. C fibre mechanical responses from intact and nerve injured rats after galanin treatment were normalised to baseline. [A] In intact naïve rats galanin and Gal2-11 were equipotent in increasing mechanical thresholds. [B] Similarly, three days after PSNI, galanin and Gal2-11 both equally increased mechanical thresholds.

Nerve injured adult rats were used three days after PSNI when the degree of mechanical allodynia had reached its peak [33] and all data below are from primary afferents that have intact receptive fields in the hindpaw ipsilateral to the injury. 100 nM galanin s.c. (*p < 0.05, baseline 7.20 ± 1.20 g vs galanin 1.20 ± 0.2 g, Figure 5A) and 100 nM Gal2-11 s.c. (**p < 0.01, baseline 4.67 ± 0.67 g vs Gal2-11 1.0 ± 0.23 g, Figure 5B) both led to a significant reduction in mechanical activation thresholds. In contrast, 10 uM galanin s.c. (**p < 0.01, baseline 7.75 ± 2.46 g vs galanin 16.50 ± 3.38 g, Figure 5C) and 10 uM Gal2-11 s.c. (*p < 0.05, baseline 3.48 ± 0.82 g vs Gal2-11 8.60 ± 2.09 g, Figure 5D) significantly increased mechanical thresholds. When normalised to baseline, data demonstrate that 10 uM galanin and 10 uM Gal2-11 are equipotent in both intact naïve (Figure 6A) and neuropathic rats (Figure 6B).

### Endogenous over-expression of galanin attenuates allodynia through actions on the primary nociceptor

**Figure 7:**
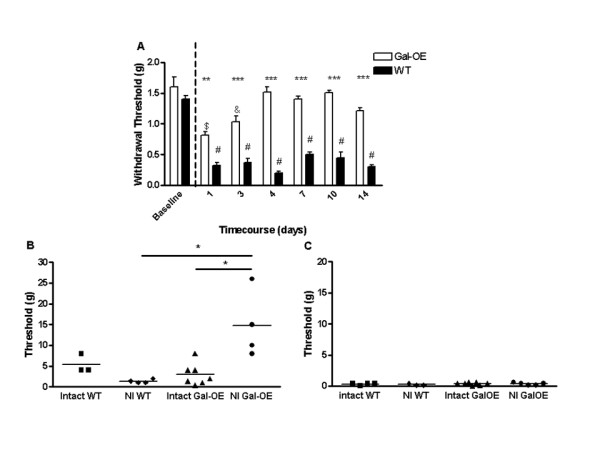
**Alterations in mechanical allodynia and primary afferent nociceptor mechanical responses in transgenic galanin over-expressing mice**. Transgenic mice that over-express galanin (Gal-OE) recover rapidly from PSNI-induced mechanical allodynia, compared to WT mice. [A] WT and Gal-OE mice have similar baseline mechanical responses before PSNI. Following surgery at day 0 (dotted line), WT mice develop robust mechanical allodynia when compared to baseline values (one way ANOVA with post-hoc Bonferroni multiple comparison test, # p < 0.001, n = 10). Gal-OE mice initially developed mechanical allodynia (one way ANOVA with post-hoc Bonferroni multiple comparison test, day 1 $ p < 0.001, day 3 & p < 0.01, n = 10) but by day 4 mechanical withdrawal thresholds had recovered to baseline values and were significantly different to WT thresholds (two way ANOVA with post-hoc Bonferroni multiple comparison test, ** p < 0.01, *** p < 0.001, n = 10,). [B] Mechanical activation thresholds of nociceptive afferents were significantly increased in the nerve injured (NI day 7) Gal-OE mice compared to uninjured Gal-OE and WT mice (one way ANOVA with post-hoc Bonferroni multiple comparison test, * p < 0.05, Intact WT n = 3, NI WT n = 4, Intact Gal-OE n = 7, NI Gal-OE n = 4). [C] The mechanical activation threshold of mechano-sensitive A fibres were unchanged in the Gal-OE mouse compared to the WT in both intact and PSNI (intact WT n = 4, NI WT n = 3, intact Gal-OE n = 7, NI Gal-OE n = 5).

We applied the PSNI model to the previously described Gal-OE and strain-matched WT mice. The Gal-OE mice over-express galanin in the DRG, spinal cord and brain under the control of the 20-kb galanin promoter [17,35] and have a 4-fold increase in the levels of galanin in the DRG (measured by radioimmunoassay) compared to WT mice, one week after axotomy [36]. Intact mice of both genotypes had similar mechanical withdrawal thresholds (Figure 7A). PSNI resulted in a rapid onset of mechanical allodynia in WT mice that was greatly attenuated in the Gal-OE. The WT mice remained allodynic for the duration of the experiment, whereas the withdrawal thresholds in the Gal-OE mice returned to baseline intact values by day 4 after PSNI and remained so for the remainder of the experiment. These behavioural findings are very similar to those previously published using the sciatic nerve spared nerve injury model of neuropathic pain [17].

To determine whether the reversal of the mechanical allodynia after PSNI in the Gal-OE mice was attributable to altered primary afferent properties, cutaneous primary mechano-sensitive afferents were studied in Gal-OE mice seven days after PSNI (when the allodynia had returned to intact baseline values) and compared to allodynic WT mice. Mechano-sensitive nociceptors (pooled C- and A delta fibres with conduction velocities (CV) of 0.33-8.5 m/s) in Gal-OE mice after PSNI had significantly higher thresholds than those from naïve Gal-OE mice (*p < 0.05, naïve Gal-OE 2.97 ± 0.99 g vs PSNI Gal-OE 14.75 ± 4.03 g, Figure 7B). In contrast, thresholds fell in WT animals after PSNI (p < 0.05, naïve WT 5.33 ± 1.33 g vs PSNI WT 1.35 ± 0.24 g, Figure 7B). No significant differences in thresholds were noted in intact naïve Gal-OE vs WT mice (Figure 7B). In addition, nociceptor spontaneous activity (PSNI WT 0.41 ± 0.25Hz vs PSNI Gal-OE 0.023 ± 0.014, p < 0.05) and the calculated afferent barrage [37,38] into the spinal cord (PSNI WT 749.7 ± 450.6Hz vs PSNI Gal-OE 41.76 ± 53.62Hz, p < 0.05) were both greatly attenuated in the injured Gal-OE mice compared to injured WT mice. In contrast to these findings, no changes in mechanical activation threshold (naïve WT 0.32 ± 0.08 g vs naïve Gal-OE 0.38 ± 0.08 g vs PSNI WT 0.24 ± 0.08 g vs PSNI Gal-OE 0.34 ± 0.08 g, Figure 7C) or spontaneous activity (naïve WT 0.1 ± 0.1Hz vs naïve Gal-OE 0.08 ± 0.04Hz vs PSNI WT 0.16 ± 0.14Hz vs PSNI Gal-OE 0.04 ± 0.21) were observed in intact injured A beta fibres (CV = 8-22 m/s).

These findings of an increase in C-fibre mechanical activation thresholds and spontaneous nociceptive responses are compatible with the observed reversal in allodynia in the Gal-OE mice, and support the hypothesis that the high levels of endogenous galanin in injured primary afferents activate a peripheral GalR2 mediated anti-nociceptive pathway.

## Discussion

The roles played by galanin and its receptor subtypes in nociception and its principal sites of action have yet to be fully defined. In this paper we have focused on the primary nociceptor and have investigated the effects of peripherally administered galanin and Gal2-11 (neither of which would be expected to cross the blood-brain-barrier) in rat and mouse, in the intact state and after the PSNI model of neuropathic pain. Our findings demonstrate that the principal effects of peripherally administered galanin are on high threshold C-fibre mechano-sensitive (HTM) primary afferents i.e. nociceptors. In intact adult animals the mechanically-evoked thresholds of nociceptive afferents is enhanced by s.c. administration of low doses of galanin or Gal2-11 directly into the receptive field, whilst at higher concentrations the mechanical thresholds of HTM afferents are greatly inhibited. This suggests that galanin has a similar dose-dependent effect on primary afferent nociceptors to those previously reported with respect to the spinal flexor reflex [12,39]. Of note, the same dose ranges of galanin s.c. were used in this study as had been previously used by Wiesenfeld-Hallin and colleagues by i.t. administration [12], and in both studies 30-300 nM galanin stimulated and 3-30 uM inhibited both nociceptor and spinal flexor reflex activity.

Having demonstrated a clear biphasic nociceptive response to peripherally administered galanin and Gal2-11 in the intact adult rodent we then used the PSNI neuropathic pain model to study the effects of peripheral administration of both ligands and transgenic over-expression of the neuropeptide on peripheral sensitisation. There is now widespread acceptance that the behavioural hypersensitivity that follows nerve injury is attributable to sensitisation of neuronal networks in the central nervous system [40-42]. In addition, it is likely that the hyperalgesia and allodynia that occurs in many neuropathic pain states is dependent, at least in part, on peripheral sensitisation, with increases in C-fibre barrage from the injured primary afferents into the spinal cord, inducing and maintaining central sensitisation [38,43,44]. We have previously shown that the PSNI model results in peripheral sensitisation of mechanically sensitive afferents in the rat and mouse, with a marked increase in spontaneous mechano-nociceptor activity [37]. Further, the spontaneous activity after PSNI in the mouse of 0.5Hz is associated with a 20-fold increase in the primary afferent barrage into the spinal cord which has previously been shown to be critical to the induction of central sensitisation [45].

Our findings in the rat after PSNI demonstrate that s.c. administration of 100 nM galanin or Gal2-11 decreased, and 10 uM increased, the mechanical activation thresholds of nociceptive afferents. The magnitude of these changes was very similar to that observed in the intact rat. Previous work by Wiesenfeld-Hallin had shown that the depressive effect of i.t. galanin on the flexor reflex occurred more often and at lower doses of galanin after sciatic nerve axotomy compared to intact rats [12]. We have not tested whether lower doses (e.g. 1 uM) of galanin or Gal2-11 would also cause a decrease in mechanically-evoked activity of nociceptive afferents in the PSNI model but not in the intact state, though one would predict that a similar finding would be observed.

The application of the PSNI model to the Gal-OE transgenic mice, which over express galanin in the DRG, spinal cord and brain, demonstrated a reversal in allodynia in the Gal-OE mice. These findings are paralleled by an abolition in nociceptor spontaneous activity and the calculated afferent barrage into the spinal cord, further supporting a peripheral anti-nociceptive role for galanin/GalR2. After nerve injury the raised levels of endogenous galanin in the DRG and in the nerve trunk following anterograde transport of galanin from the cell body to the site of injury [46], could exert a direct inhibitory effect on primary afferent fibres via activation of GalR2, leading to a marked reduction in spontaneous neuronal firing and C-fibre barrage into the spinal cord and thus an attenuation in neuropathic pain behaviour. At present we are unable to separate the effects of the galanin overexpression in the DRG of these transgenic mice from that in the spinal cord and/or brain. It is only when galanin drug-like agonists are characterized that do or do not penetrate the blood-brain barrier, that their specific sites of action can be systematically tested.

In the present study we have shown that the biphasic nociceptive responses to peripherally administered galanin and the GalR2/3-specific ligand Gal2-11 (which does not activate GalR1 [26]) in intact and nerve injured rats are almost identical. Further, levels of GalR3 mRNA expression in the DRG and dorsal horn are undetectable by in-situ hybridization [25] and only barely detectable in the DRG using RT-PCR [21], and do not appear to change after peripheral nerve injury [21]. These findings imply that the above effects of galanin and Gal2-11 are most likely to be mediated by activation of GalR2. However further investigation into the relative involvement of GalR1 and GalR2 in nociception are not possible at present due to the current lack of drug-like pharmacological tools that are GalR1- and GalR2-specific.

To date, the majority of published work using the GalR2/3 specific agonist Gal2-11 has shown that central GalR2 activation plays a predominantly stimulatory role with regard to nociception. Intrathecal infusion of low dose Gal2-11 in the intact rat reduces mechanosensory thresholds [26] and peripheral administration of the agonist in the low nanomolar range into the peripheral receptive field facilitates inflammatory pain behaviours [47]. Consistent with this, ~70% of dispersed adult rat sensory neurons in culture responded to galanin and Gal2-11, via coupling to G_q/11 _which leads to an increase in calcium influx and neuronal excitability [18]. These results have led many to hypothesise that activation of GalR1 is inhibitory and plays an anti-nociceptive role whilst GalR2 is excitatory and pro-nociceptive [48]. More recently however, further data has shown that GalR2 activation may also be inhibitory with regard to nociception. Smith and colleagues have shown that activation of GalR2 in the presynaptic terminals of the dorsal horn of the spinal cord can lead to the inhibition of sensory neuron excitability, compatible with an anti-nociceptive role for the receptor [49]. Similarly, work by Wiesenfeld-Hallin and colleagues has shown that i.t. administration of micromolar doses of galanin robustly inhibited windup and reflex facilitation in intact GalR1-KO mice, confirming that GalR2 activation in the spinal cord can also be inhibitory with respect to nociception [50]. This conclusion is further strengthened by recent work by the Stebbing lab demonstrating that GalR2 activation directly inhibits calcium currents in dispersed cultured DRG neurons [51] and provides a precedented mechanism of action by which GalR2 activation in the DRG may reduce nociception.

The mechanism(s) by which activation of GalR2 by low dose galanin or Gal2-11 facilitates nociceptor and spinal flexor reflex activity and inhibits these same neurons at higher doses are unclear. Studies using in-situ hybridization (there are no antisera specific for either receptor subtype that can be used for immunocytochemistry) have shown that the majority of the neurons in the DRG express both GalR1 and GalR2 mRNAs [18] whilst in the dorsal horn GalR1 expression predominates [22]. It is possible therefore that the observed dose-dependent responses to galanin and Gal2-11 might be explained in part by differing responses of subpopulations of neurons that express varying levels of GalR1 or GalR2. However, our findings that galanin and the GalR2/3 agonist Gal2-11 have very similar dose-dependent actions on mechanosensory thresholds in the intact animals and after nerve injury makes this explanation less likely, and implies the dual effects are both mediated by activation of GalR2. It is now well established that GalR2 couples both to G_i _(inhibiting adenylyl cyclase and thus nociception) and to G_q/11 _(which in turn activates MAPK and Akt, reviewed in [52], and thus may play a pro-nociceptive role). The kinetics of these two pathways in the DRG and the spinal cord are unknown and it is possible that the G_q/11 _pathway "saturates" and reaches maximum before G_i_. Thus as the dose of galanin or Gal2-11 is increased the inhibitory downstream signalling pathway becomes predominant explaining why both ligands are anti-nociceptive at micromolar concentrations. Evidence for opposing G-protein regulated transduction pathways with differing concentrations of ligands in GPCRs, comes from work by Teschemacher who showed that low doses of angiotensin II inhibit exocytosis in a G_i_-dependent manner in chromaffin cells, whilst high concentrations of ligand potentiate catecholamine release via activation of PLC and calcium mobilisation from internal stores [53]. Similarly, low doses of agonist activate beta-2 adrenergic receptors through Gα_s _whilst high concentrations are transduced via a G-protein-independent pathway that appears to require the Src tyrosine kinase [54]. Both of these papers are analogous to our current data and that of Wiesenfeld-Hallin, and again support the hypothesis that the observed dose-dependent agonist effects on nociception are mediated by GalR2 via different intracellular signalling pathways.

Our current findings and those of Wiesenfeld-Hallin raise the issue of whether the local extra-cellular levels of galanin after nerve injury are sufficient to inhibit nociceptive responses via activation of GalR1 and/or GalR2. Previous work by Duggan et al using antibody microprobes has shown an extensive basal release of galanin in the dorsal horn of intact adult rats, with a significant rise in release in the superficial layer of the cord ipsilateral to peripheral nerve injury [55,56]. These studies have not been extended to the DRG nor was quantitation of the absolute extra-cellular levels of galanin possible using the antibody microprobe technique. In the current study and that of Wiesenfeld-Hallin, micromolar amounts of galanin were injected s.c. into the paw or by bolus i.t. injection into the lumbar enlargement respectively, and in both cases the local concentration at the receptor terminals will inevitably have been reduced by a combination of local tissue dilution and degradation.

In summary, we have demonstrated yet another layer of complexity in the "galanin pain story". Our data supports the hypothesis that peripheral activation of GalR2 by the raised levels of endogenous galanin in injured primary afferents increases C-fibre thresholds and decreases evoked and spontaneous activity. These changes in primary afferent properties lead to a decrease in C-fibre barrage into the spinal cord which would be expected to reduce central sensitisation and allodynia. These findings will need to be further substantiated in intact rodents and the neuropathic pain models described above, once GalR2-specific drug-like agonists are identified and characterized. Of note, the previously described loss of a subset of small diameter unmyelinated neurons that are likely to be nociceptors in the DRG of Gal-KO [57] and GalR2-KO mice [21], precludes the use of these mice to further analyse the nociceptive role played by GalR2 in the adult animal. Similarly, immunoneutralising antisera against galanin that can be used in-vivo have yet to be described.

## Materials and methods

Experiments were performed on 8 week old male Wistar rats and 8 week old male Gal-OE and strain matched wildtype (WT) CBA/Bl6 mice [17,36]. Animals were fed standard chow and water ad libitum and all experiments were carried out in accordance with the United Kingdom Animals (Scientific Procedures) Act 1986. In accordance with UK legislation, in all experiments the minimum numbers of animals were used to achieve statistical significance.

Partial saphenous nerve ligation injury (PSNI) was performed as previously described [33,34,37]. Anaesthesia was induced and maintained with either isoflurane or halothane in O_2 _(3% induction, 2% maintenance). An incision was made in the inguinal fossa of the right hind leg exposing the superficial saphenous nerve. The saphenous nerve was isolated proximal to any branch points using blunt dissection with fine forceps from the surrounding tissues. PSNI surgery prior to electrophysiogical experiments had to be positioned to allow sufficient nerve proximal to the injury site to allow recordings to be made. A size 4.0 sterile silk suture in rat (size 7.0 in mice) was used to ligate ~50% of the total nerve, with the lateral half of the nerve being ligated for consistency. The skin was sutured with a 4.0 sterile silk suture. Animals were monitored and allowed to recover thereafter for the duration of the experiments. Electrophysiological experiments were carried out three days after PSNI in rat and seven days in mice.

### Electrophysiology

Anaesthesia was induced with sodium pentobarbital (60 mg/kg i.p. rats, 10 mg/kg i.p. mice, Sigma-Aldrich, UK) and were then maintained deeply anaesthetised and areflexive using continuous infusion of sodium pentobarbital (~20 mg/kg/hr i.v. via an external jugular cannula in rat, and ~2 mg/kg/hr i.p. mice). Body temperature was maintained within physiological limits by means of a feedback controlled heater and rectal thermister. At the end of all experiments, rats and mice were killed by an overdose of sodium pentobarbital.

An incision was made along the right inguinal fossa to expose the saphenous nerve. Using blunt dissection, skin was freed from surrounding tissue and this was used to create a pool. To keep the tissue hydrated the pool was filled with warmed mineral oil. In the mouse, the pool was lined with Xantopren XL, which set once mixed with Activator Universal (Heraeus Kulzer, Germany). This prevented mineral oil leakage, due to the porosity of mouse skin. The saphenous nerve was exposed and the epineurium was removed to allow fine filaments to be dissected from the main trunk of the nerve. Filaments were placed on bipolar platinum recording electrodes for differential recordings of neuronal activity to identify individual afferent fibres. After nerve injury, recordings were made proximal to the site of injury. This recorded activity was amplified, filtered and captured for off-line analysis by a micro1401 (Cambridge Electronic Design, U.K) and P.C interface (Viglen) using Spike 2 software (C.E.D. Cambridge, UK).

A search protocol was used to identify afferents that innervated the hindpaw (no higher than the ankle) and had identifiable receptive fields. Initial characterisation of afferents involved mechanical search stimulus (brush/glass rod/probe/pinch with blunt forceps) and monopolar electrical search stimulation. The mechanical search stimulus was used to apply pressure to discrete areas of the foot to stimulate robust responses from mechanically sensitive afferents. Electrical stimulation involved the use of a monopolar (cathode) electrode. The monopolar electrode was used to determine conduction velocity (CV) for fibres in m/s. This was applied to the receptive field on the hindpaw (0.5 ms duration, at max. 100V intensity, rate 0.3Hz). The CV for afferents were defined as C-fibres <1 m/s, and A-fibres as >1 m/s [58,59]. These stimuli allowed the identification and characterisation of individual mechano-sensitive afferents. To ensure that recordings acquired were from one individual afferent, afferents were teased to ensure that there were minimal numbers of fibres with receptive fields in the hindpaw and that there was minimal overlap of these receptive fields. Offline sorting analysis of waveforms using Spike 2 software (C.E.D.) confirmed responses to be from an individual afferent fibre.

Once a single primary afferent receptive field had been identified, it was further characterised. Brush, pinch with blunt forceps and the use of direct calibrated force with von Frey hairs (Linton instruments, UK) were all applied to the receptive field to determine mechanical response properties. Mechanical thresholds were identified by applying a range of von Frey (vF) hairs, with each vF applied no more than three times to the receptive field for 5 seconds as previously described [58,60-62]. Using an up down method, the lowest force that reproducibly elicited robust firing (firing >3 action potentials) of the identified afferent fibre was determined to be the mechanical threshold [59,63]. Nociceptive (or high threshold mechano-sensitive (HTM)) afferents responded to pinch when applied to the receptive field but not brush, typically von Frey activation thresholds were above 1 g [33,58]. In the mouse, nociceptive afferents (were not brush sensitive) were derived from pooled C- and A-delta fibre groups. Mechanically evoked activity was also recorded by applying a range of von Frey hairs (rat; 0.4 g-180 g mouse; 0.16-2 g) to the receptive field for 5 seconds, each hair being applied three times. Following the mechanical characterisation of the afferent, these responses were also recorded after vehicle and drug administration. The operator was blinded to mouse genotype throughout the experiments.

Mechano-nociceptor afferent activation threshold data in intact naïve and PSNI groups after galanin or Gal2-11 injections were normalised to the respective mean baseline thresholds, allowing direct comparisons between the groups. The total primary afferent barrage was calculated as previously described [37], from the numbers of unmyelinated afferents in the saphenous nerve [64,65]. This value was multiplied by the mean value of ongoing activity and if the nerve was injured, the value was divided by two to represent the 50% of the saphenous nerve left intact.

### Drug administration

Vehicle and drugs were administered using an insulin needle (BD micro fineTM). 30ul subcutaneous injections (s.c.) were administered just outside the identified receptive field. The initial s.c. injection involved injecting 30ul of vehicle (saline) thirty minutes after characterisation of the afferents. Five minutes later mechanical thresholds and evoked activity were recorded. If mechanical activation thresholds and/or mechanical evoked activity were significantly diminished by the vehicle injection (implying local tissue damage or inflammation, resulting in changes in afferent properties), the effect of the drug was not investigated. If afferent properties were unchanged by vehicle then the drug was injected at the same site thirty minutes after vehicle injection. Mechanical thresholds and evoked activity were determined at one and/or five minutes after injection. At one minute, only the activation threshold was identified due to the time constraints of the experiment. Different concentrations of galanin (rat, Bachem UK) and the GalR2/3 agonist Gal2-11 (Sigma-Aldrich, UK) were administered. Repeated vehicle (saline) injections showed no change in mechanical activation threshold (data not shown).

### Behavioural Testing

The day prior to behavioural testing mice were placed in transparent perspex enclosures and habituated to the testing environment for a minimum of 15 minutes until they settled. Habituation occurred prior to all behavioural testing sessions. Mechanical withdrawal thresholds were recorded from both ipsilateral and contralateral hind paws. Mechanical withdrawal thresholds were determined using a series of calibrated von Frey filaments (Linton Instruments, UK). Each von Frey hair ranging from 0.16 g to 2 g was applied to the medial plantar surface of each hindpaw 5 times for a maximum period of 5 seconds or until a withdrawal was elicited. The 50% mechanical withdrawal thresholds (the force at which the animal withdrew the paw 50% of the time), were then calculated from stimulus/response curves, generated as previously described [33,37,58].

### Statistical Analysis

Data are shown as mean ± SEM. Mechanically evoked activity before and after drug along with mechanical withdrawal testing were compared using one way ANOVA with post hoc Bonferroni tests (* p < 0.05, ** p < 0.01, *** p < 0.001). Activation thresholds were compared using either one-way ANOVAs with post hoc Bonferroni multiple comparison test or paired T test (* p < 0.05, ** p < 0.01, *** p < 0.001).

## Competing interests

The authors declare that they have no competing interests.

## Authors' contributions

RH carried out nociceptive behaviour and teased fibre electrophysiological experiments. RH, LFD and DW were responsible for experimental design and drafting of the manuscript. All authors have read and approved this final version of the manuscript.
